# Bosutinib for pretreated patients with chronic phase chronic myeloid leukemia: primary results of the phase 4 BYOND study

**DOI:** 10.1038/s41375-020-0915-9

**Published:** 2020-06-22

**Authors:** Andreas Hochhaus, Carlo Gambacorti-Passerini, Camille Abboud, Bjørn Tore Gjertsen, Tim H. Brümmendorf, B. Douglas Smith, Thomas Ernst, Pilar Giraldo-Castellano, Ulla Olsson-Strömberg, Susanne Saussele, Nathalie Bardy-Bouxin, Andrea Viqueira, Eric Leip, T. Alexander Russell-Smith, Jocelyn Leone, Gianantonio Rosti, Justin Watts, Francis J. Giles, on behalf of the BYOND Study Investigators, E. Abruzzese, L. P. Akard, A. Bosi, F. Cervantes, A. Charbonnier, F. Di Raimondo, G. Etienne, V. Garcia Gutierrez, A. P. Guerci-Bresler, H Hjorth-Hansen, J. M. Karsenti, K. R. Kelly, P. Le Coutre, C. Martinez Chamorro, V. G. Oehler, G. Orti Pascual, A. Petzer, E. Pungolino, G. Rege-Cambrin, F. Rigal-Huguet, G. J. Roboz, P. Rousselot, F. M. Sanchez-Guijo, G. Sanz Santillana, P. Schafhausen, C. Scheid, S. Schmidt, G. Specchia, J. L. Steegmann, L. Stenke

**Affiliations:** 1 Klinik für Innere Medizin II, Universitätsklinikum Jena, Jena, Germany; 2 University of Milano-Bicocca, Monza, Italy; 3 Washington University School of Medicine, St. Louis, MO USA; 4 Haukeland University Hospital, Helse Bergen, and University of Bergen, Bergen, Norway; 5 Universitätsklinikum RWTH Aachen, Aachen, Germany; 6 Johns Hopkins Sidney Kimmel Comprehensive Cancer Center, Baltimore, MD USA; 7 CIBER Enfermedades Raras, Miguel Servet University Hospital, Zaragoza, Spain; 8 University of Uppsala and Department of Hematology, University Hospital, Uppsala, Sweden; 9 Universitätsmedizin Mannheim, Heidelberg University, Mannheim, Germany; 10 Pfizer International Operation-Oncology, Paris, France; 11 Pfizer SLU, Madrid, Spain; 12 Pfizer Inc, Cambridge, MA USA; 13 Pfizer Inc, New York, NY USA; 14 University Hospital, University of Bologna, Bologna, Italy; 15 University of Miami, Sylvester Comprehensive Cancer Center, Miami, FL USA; 16 Developmental Therapeutics Consortium, Chicago, IL USA; 17 Ospedale S. Eugenio, Rome, Italy; 18 Indiana Blood and Marrow Transplantation-Clinic, Indianapolis, IN USA; 19 SOD Ematologia, Firenze, Italy; 20 Hospital Clinic IDIBAPS, Barcelona, Spain; 21 Centre Regional De Lutte Contre Le Cancer, Marseille, France; 22 Policlinico Vittorio Emanuele, Catania, Italy; 23 Institut Bergonie, Bordeaux, France; 24 Hospital Universitario Ramon y Cajal, Madrid, Spain; 25 CHU Brabois, Vandoeuvre-les-Nancy, France; 26 St Olav Hospital, Trondheim, Norway; 27 CHU Nice-Hopital Archet, Nice, France; 28 University of Southern California, Norris Comprehensive Cancer Center, Los Angeles, CA USA; 29 Charité – Universitätsmedizin Berlin, Berlin, Germany; 30 Hospital Universitario Quiron de Madrid, Pozuelo de Alarcon, Madrid, Spain; 31 Seattle Cancer Care Alliance, Seattle, WA USA; 32 Vall dʼHebron University Hospital, Barcelona, Spain; 33 Ordensklinikum Linz Gmbh Barmherzige Schwestern, Linz, Austria; 34 Ospedale Niguarda Ca Granda, Milano, Italy; 35 San Luigi Gonzaga SCDU, Orbassano, Italy; 36 Institut Universitaire du Cancer Toulouse, Toulouse, France; 37 Weill Cornell Medical College - New York-Presbyterian Hospital, New York, NY USA; 38 Centre Hospitalier de Versailles, Hopital Andre Mignot, Le Chesnay, France; 39 Hospital Clinico Universitario de Salamanca, Salamanca, Spain; 40 Hospital Universitari i Politecnic La Fe, Valencia, Spain; 41 Universitätsklinikum Hamburg-Eppendorf, Hamburg, Germany; 42 Universitätsklinikum Köln, Köln, Germany; 43 Medizinische Universität Innsbruck, Innsbruck, Austria; 44 Policlinico Consorziale di Bari, Bari, Italy; 45 Hospital Universitario de La Princesa, Madrid, Spain; 46 Hematologiskt Centrum, Stockholm, Stockholm, Sweden

**Keywords:** Drug development, Chronic myeloid leukaemia

## Abstract

Bosutinib is approved for newly diagnosed Philadelphia chromosome-positive (Ph+) chronic phase (CP) chronic myeloid leukemia (CML) and for Ph+ CP, accelerated (AP), or blast (BP) phase CML after prior treatment with tyrosine kinase inhibitors (TKIs). In the ongoing phase 4 BYOND study (NCT02228382), 163 CML patients resistant/intolerant to prior TKIs (*n* = 156 Ph+ CP CML, *n* = 4 Ph+ AP CML, *n* = 3 Ph-negative/*BCR-ABL1*+ CML) received bosutinib 500 mg once daily (starting dose). As of ≥1 year after last enrolled patient (median treatment duration 23.7 months), 56.4% of Ph+ CP CML patients remained on bosutinib. Primary endpoint of cumulative confirmed major cytogenetic response (MCyR) rate by 1 year was 75.8% in Ph+ CP CML patients after one or two prior TKIs and 62.2% after three prior TKIs. Cumulative complete cytogenetic response (CCyR) and major molecular response (MMR) rates by 1 year were 80.6% and 70.5%, respectively, in Ph+ CP CML patients overall. No patient progressed to AP/BP on treatment. Across all patients, the most common treatment-emergent adverse events were diarrhea (87.7%), nausea (39.9%), and vomiting (32.5%). The majority of patients had confirmed MCyR by 1 year and MMR by 1 year, further supporting bosutinib use for Ph+ CP CML patients resistant/intolerant to prior TKIs.

## Introduction

Chronic myeloid leukemia (CML) is a myeloproliferative neoplasm characterized by the presence of the Philadelphia chromosome (Ph) [1]. Imatinib was the first BCR-ABL1-targeting tyrosine kinase inhibitor (TKI) approved for the treatment of CML [2, 3]. The 2nd-generation TKIs dasatinib, nilotinib, and bosutinib can be used as first-line therapy alternatives to imatinib for chronic phase (CP) CML [4–6]. However, patients may become resistant or intolerant to first-line TKI treatment [7–10]. Therapy options in the second-line setting are dasatinib, nilotinib, and bosutinib, or the 3rd-generation BCR-ABL1 TKI ponatinib [11–14]. TKIs radotinib and asciminib are emerging as treatment options [9, 15].

Approval of bosutinib for patients with Ph+ CP, accelerated phase (AP), or blast phase (BP) CML previously treated with ≥1 TKI was based on results from a phase 1/2 study [13, 16]. In patients with imatinib-resistant or imatinib-intolerant Ph+ CML, and in patients who had received prior imatinib plus dasatinib and/or nilotinib, bosutinib 500 mg once daily (QD) demonstrated durable efficacy and manageable toxicity after longer follow-up [17–19]. At year 5, 40% of patients resistant/intolerant to imatinib remained on bosutinib; cumulative major cytogenetic response (MCyR), complete cytogenetic response (CCyR), and major molecular response (MMR) rates were 60%, 50%, and 42%, respectively [19]. In a 4-year follow-up of patients receiving bosutinib in the third- or fourth-line setting, cumulative MCyR and CCyR rates were 40% and 32%, respectively [18]. Across all patients with Ph+ CP CML in that study, the most common (≥30%) adverse events (AEs) were diarrhea, nausea, and vomiting [18, 19].

The purpose of the current phase 4 study was to provide further information on the treatment with bosutinib of patients with CML resistant/intolerant to prior TKIs or who were otherwise not appropriate for treatment with other TKIs. This study also aimed to fulfill a post-authorization commitment to the European Medicines Agency regarding the efficacy and safety of bosutinib in this patient population.

## Methods

### Study design and patients

BYOND (NCT02228382) is an ongoing, single-arm, open-label, non-randomized phase 4 study of bosutinib in patients with chronic or advanced Ph+ CML who have failed prior treatment with TKIs. Eligible patients were adults with a cytogenetic or qualitative polymerase chain reaction-based diagnosis of Ph+ and/or *BCR-ABL1*+ CML (from initial diagnosis), prior treatment with ≥1 TKI for CML and adequate hepatic/renal function. Any CML phase was permitted, as long as the patient was resistant/intolerant to prior TKIs. Patients with CP CML and treated with one or two prior TKIs were required to have Eastern Cooperative Oncology Group performance status (ECOG PS) 0 or 1; those with CP CML after three prior TKIs and with AP/BP CML could have ECOG PS 0–3. Patients with leptomeningeal leukemia or a known *BCR-ABL1* T315I or V299L mutation were excluded. Additional details on eligibility criteria are in Supplementary Methods.

Patients received bosutinib at a starting dose of 500 mg QD. Dose escalation to a maximum of 600 mg QD was permitted due to unsatisfactory response or signs of disease progression in the absence of any grade 3/4 or persistent grade 2 AEs. Dose reduction to 400, 300, or 200 mg QD due to toxicity/tolerability was permitted (see Supplementary Methods**)**. Patients were to receive bosutinib for up to 4 years from the time of first dose, unless disease progression, unacceptable toxicity, withdrawal of consent, death, or study discontinuation. Patients who discontinued bosutinib prior to completing 4 years of therapy were to be followed for survival until they completed 4 years on study.

The study was approved by institutional review boards and independent ethics committees at each center. The study was conducted in accordance with all local legal and regulatory requirements, as well as the general principles set forth in the International Ethical Guidelines for Biomedical Research Involving Human Patients, Guidelines for Good Clinical Practice and the Declaration of Helsinki. All patients provided written informed consent.

### Endpoints and analyses

The primary endpoints were cumulative confirmed MCyR (in two consecutive analyses ≥28 days apart) by 1 year (52 weeks) in patients with Ph+ CP CML treated with one or two prior TKIs and three prior TKIs, and cumulative confirmed overall hematologic response (OHR; in two consecutive analyses ≥28 days apart) by 1 year (52 weeks) in patients with AP or BP CML. Cumulative confirmed MCyR was defined as CCyR (0% Ph+ from ≥20 metaphases or <1% fluorescent in situ hybridization [FISH] positive cells from ≥200 interphase nuclei) or partial cytogenetic response (PCyR; >0%, ≤35% Ph+). To be considered a responder, the patient must have had maintenance of baseline response for ≥52 weeks for cytogenetic response or an improvement from baseline. Patients with PCyR at baseline must have attained CCyR on-treatment to be considered a cytogenetic responder. Patients with at least MMR and a deeper molecular response (MR) than baseline were counted as confirmed CCyR. Cumulative confirmed OHR was defined as complete hematologic response (CHR) or return to CP.

Key secondary and exploratory endpoints included: cumulative MCyR (unconfirmed); cumulative MMR (*BCR-ABL1* International Scale [IS] ≤ 0.1%), MR^4^ (*BCR-ABL1* IS ≤ 0.01%), and MR^4.5^ (*BCR-ABL1* IS ≤ 0.0032%); *BCR-ABL1* mutational analyses; on-treatment transformation to AP or BP CML; overall survival (OS); safety; and patient-reported outcome (PRO) measures.

Analyses for molecular, cytogenetic, and hematologic responses are described in Supplementary Methods. CCyR was imputed from MMR on a specific date if there was no valid cytogenetic assessment. Time to response was defined as the interval from the date of first dose of bosutinib to initial response. Non-responders were censored at the last valid assessment date for the respective endpoint. OS was defined as the interval from the date of first dose of bosutinib to the date of death due to any cause. Patients not known to have died were censored at the last known alive date. Time to response was estimated using cumulative incidence, adjusting for the competing risk of treatment discontinuation without the event; OS was estimated using the Kaplan–Meier method. Two-sided 95% confidence interval (CI) for response rate was determined using the exact binomial method. For Kaplan–Meier's yearly probability estimates, two-sided 95% CI was based on Greenwood’s formula using a log(-log) transformation.

Treatment-emergent AEs (TEAEs), serious AEs, and laboratory evaluations were assessed up to 28 days after last dose. Events were graded according to the National Cancer Institute Common Terminology Criteria for Adverse Events, v4.0. The frequency of selected adverse events of special interest was analyzed by selecting Medical Dictionary for Regulatory Activities (MedDRA) system organ class higher level group, higher level and preferred terms and standardized MedDRA queries to generate TEAE clusters (see Supplementary Methods).

PROs were assessed using the Functional Assessment of Cancer Therapy-Leukemia (FACT-Leu) quality-of-life (QoL) questionnaire (see Supplementary Methods). For each cohort at each of the timepoints, summary statistics for the observed values as well as changes from baseline were estimated. As a supplemental post hoc analysis, a repeated measures longitudinal model was used to estimate the relationship between MR (screening to month 12 represented by a log-reduction scale) as a predictor and FACT-Leu total score and each domain score as an outcome. The standardized effect sizes were calculated to determine strength of effects and allow comparisons across FACT-Leu domains.

This study did not include any formal sample size determination and results are descriptive only. Approximately 150 patients with Ph+ CML were to be enrolled, including ≥45 patients with CP, AP, or BP CML treated in the fourth-line or later setting. All treated patients with Ph+ CML with a valid baseline efficacy assessment for the respective endpoint (evaluable population) were included in the molecular, cytogenetic, and hematologic efficacy analyses. All patients who received ≥1 dose of study drug (full analysis set) were included in the safety analyses and those with Ph+ CP CML were included in the PRO analyses. Ph+ CP CML patients were also analyzed by resistance or intolerance to prior TKIs as assessed by the investigator (Supplementary Methods). Data are from an unlocked trial database with a cut-off date of September 18, 2018, ≥12 months after last enrolled patient.

## Results

### Patients and treatment

A total of 163 patients were enrolled between November 20, 2014 and September 18, 2017 across 41 study centers in eight countries. Of 163 patients who received bosutinib, 156 had Ph+ CP CML, four had Ph+ AP CML, and three had Ph−/*BCR-ABL1*+ CP CML. Across Ph+ CP CML cohorts, 51.9% of patients were male and median age was 61.0 years; 29.5%, 39.1%, and 31.4% received bosutinib as second-, third- and fourth-line TKI therapy, respectively (Table 1). In all, 53.2% of patients with Ph+ CP CML were resistant to ≥1 prior TKI and 46.8% were intolerant to all prior TKIs. Imatinib was the most common prior TKI, received by 90.4% of patients. All patients with AP CML were male, with a median age of 40.0 years; two each received bosutinib as third- and fourth-line TKI therapy. All patients with Ph− CML were male, with a median age of 63.0 years; two received bosutinib as second-line and one as third-line TKI therapy.

**Table 1:** Demographic and baseline characteristics across patients with Ph + CP CML.

Characteristic	Line of treatment			Total (*N* = 156)
	Second-line (*n* = 46)	Third-line (*n* = 61)	Fourth-line (*n* = 49)	
Male, *n* (%)	23 (50.0)	37 (60.7)	21 (42.9)	81 (51.9)
Age, median (range), years	54.0 (19.0–88.0)	65.0 (27.0–85.0)	61.0 (21.0–85.0)	61.0 (19.0–88.0)
Age group, *n* (%)				
<65 years	34 (73.9)	30 (49.2)	32 (65.3)	96 (61.5)
≥65 years	12 (26.1)	31 (50.8)	17 (34.7)	60 (38.5)
ECOG PS, *n* (%)				
0	34 (73.9)	40 (65.6)	32 (65.3)	106 (67.9)
1	12 (26.1)	20 (32.8)	13 (26.5)	45 (28.8)
2	0	1 (1.6)	4 (8.2)	5 (3.2)
Median (range) duration since CML diagnosis, years	2.2 (0.2–11.4)	5.0 (0.3–18.6)	7.3 (0.7–27.7)	4.7 (0.2–27.7)
Prior TKI, *n* (%)^a^				
Imatinib	35 (76.1)	57 (93.4)	49 (100)	141 (90.4)
Dasatinib	5 (10.9)	41 (67.2)	49 (100)	95 (60.9)
Nilotinib	6 (13.0)	24 (39.3)	49 (100)	79 (50.6)
Prior interferon alpha, *n* (%)	2 (4.3)	3 (4.9)	6 (12.2)	11 (7.1)
Resistant to any prior TKI, *n* (%)	17 (37.0)	35 (57.4)	31 (63.3)	83 (53.2)
Intolerant to all prior TKIs, *n* (%)	29 (63.0)	26 (42.6)	18 (36.7)	73 (46.8)

Full analysis set.

*CML* chronic myeloid leukemia, *CP* chronic phase, *ECOG PS* Eastern Cooperative Oncology Group performance status, *Ph* Philadelphia chromosome, *TKI* tyrosine kinase inhibitor.

^a^In the third-line cohort, 37 (60.7%) of patients received prior imatinib and dasatinib, 20 (32.8%) of patients received prior imatinib and nilotinib and 4 (6.6%) of patients received prior dasatinib and nilotinib.

**Fig. 1:**
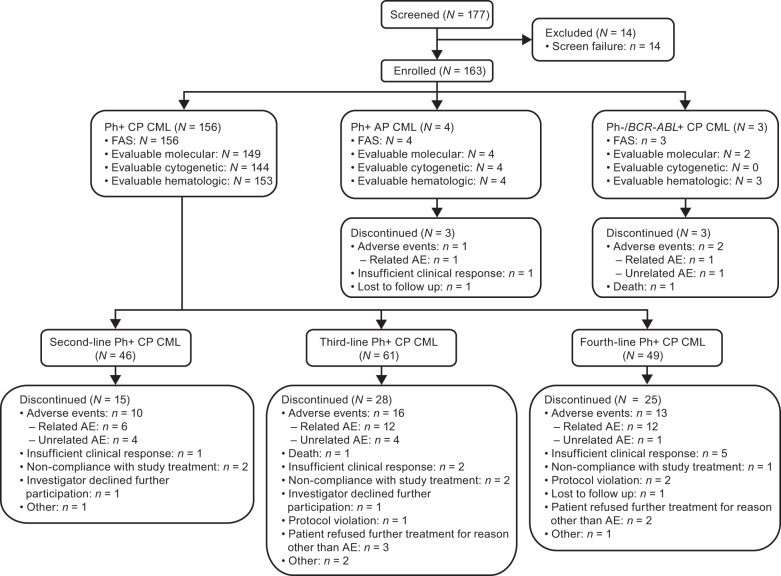
Patient disposition. Full analysis set. The 14 participants screened but not enrolled did not meet the eligibility criteria. *AE* adverse events, *AP* accelerated phase, *CML* chronic myeloid leukemia, *CP* chronic phase, *FAS* full analysis set, *Ph* Philadelphia chromosome.

As of ≥1 year after last enrolled patient (~85% with ≥2-year follow-up), 56.4% of patients with Ph+ CP CML remained on bosutinib: 67.4%, 54.1%, and 49.0% in the second-, third-, and fourth-line cohorts, respectively (Fig. 1). In all, 59.0% of TKI-resistant patients and 53.4% of TKI-intolerant patients remained on bosutinib. The most common primary reasons for permanent treatment discontinuation were AEs in 39 (25.0%) and insufficient clinical response in eight (5.1%) patients. Three of four patients with AP CML discontinued bosutinib due to AE, insufficient response, or lost to follow-up (*n* = 1 each). All three patients with Ph− CP CML discontinued bosutinib due to AE (*n* = 2) or death (*n* = 1).

Median (range) duration of bosutinib treatment was 23.7 (0.2–42.2) months in the Ph+ CP CML cohort: 25.9 (0.9–41.2), 24.2 (0.4–42.2), and 12.3 (0.2–41.9) in the second-, third-, and fourth-line cohorts, respectively. In the AP and Ph− CML cohorts, median (range) duration of bosutinib treatment was 18.0 (1.6–32.3) and 7.2 (3.4–25.8) months, respectively. Median (range) dose intensity in the Ph+ CP CML cohort was 313.1 (79.7–560.6) mg/day: 320.1 (98.4–560.6), 309.4 (79.7–500.0), and 308.0 (125.0–500.0) in the second-, third-, and fourth-line cohorts, respectively. In the AP and Ph− CML cohorts, median (range) dose intensity was 497.9 (346.6–500.0) and 296.2 (81.0–422.8) mg/day, respectively. Median (range) duration of bosutinib treatment was 23.4 (0.2–42.2) months in TKI-resistant and 25.3 (0.4–41.9) months in TKI-intolerant patients, respectively. Corresponding median (range) dose intensity was 405.9 (125.0–560.6) and 292.0 (79.7–500.0) mg/day. At all timepoints, 500 mg QD was the most commonly utilized dosage and >50% of patients with Ph+ CP CML were receiving 400 or 500 mg QD (Supplementary Fig. S1).

### Efficacy

Of 144 evaluable patients with Ph+ CP CML, the primary endpoint of cumulative confirmed MCyR rate (95% CI) by 1 year was 75.8% (66.1–83.8%) in those treated with one or two prior TKIs, and 62.2% (46.5–76.2%) in those previously treated with three TKIs. In all, 112 (77.8%) patients with Ph+ CP CML had MCyR at baseline. In the overall Ph+ CP CML cohort, the cumulative confirmed MCyR rate (95% CI) by 1 year was 71.5% (63.4–78.7%); 64.6% of patients achieved a deeper response relative to baseline and 6.9% maintained their baseline response for ≥1 year. In the four patients with AP CML, the primary endpoint of cumulative confirmed OHR rate (95% CI) by 1 year was 75.0% (19.4–99.4%), as was cumulative confirmed CHR rate.

Cumulative MCyR and CCyR rates, respectively, by 1 year were 83.3 and 80.6% in patients with Ph+ CP CML (TKI-resistant: 79.2 and 75.3%; TKI-intolerant: 88.1 and 86.6%); cumulative MCyR and CCyR rates by line of therapy are shown in Table 2. In patients without the respective baseline response, cumulative MCyR and CCyR rates, respectively, by 1 year were 59.4 and 63.5% (TKI-resistant: 56.5 and 58.8%; TKI-intolerant: 66.7 and 72.2%; Table 2). Cytogenetic responses were achieved within 1 year with the exception of one TKI-resistant patient who achieved a CCyR after month 12.

**Table 2:** Cumulative cytogenetic response rates by 1 year in patients with Ph + CP CML: total cohort, by line of therapy, and by TKI resistance or intolerance (overall and excluding patients with the respective baseline response).

	Total *N* = 156	By line of therapy			By TKI resistance or intolerance	
		Second-line *n* = 46	Third-line *n* = 61	Fourth-line *n* = 49	Resistant *n* = 83	Intolerant *n* = 73
Cumulative cytogenetic response, % (95% CI)						
Evaluable patients, *n*	144	43	56	45	77	67
MCyR	83.3 (76.2–89.0)	88.4 (74.9–96.1)	83.9 (71.7–92.4)	77.8 (62.9–88.8)	79.2 (68.5–87.6)	88.1 (77.8–94.7)
CCyR	80.6 (73.1–86.7)	83.7 (69.3–93.2)	83.9 (71.7–92.4)	73.3 (58.1–85.4)	75.3 (64.2–84.4)	86.6 (76.0–93.7)
Cumulative cytogenetic response in patients without the respective baseline response, % (95% CI)						
Evaluable patients, *n*	32	10	10	12	23	9
MCyR	59.4 (40.6–76.3)	80.0 (44.4–97.5)	60.0 (26.2–87.8)	41.7 (15.2–72.3)	56.5 (34.5–76.8)	66.7 (29.9–92.5)
Evaluable patients, *n*	52	16	19	17	34	18
CCyR	63.5 (49.0–76.4)	75.0 (47.6–92.7)	68.4 (43.4–87.4)	47.1 (23.0–72.2)	58.8 (40.7–75.4)	72.2 (46.5–90.3)

Evaluable cytogenetic population. To be considered a responder, the patient must have maintenance of baseline response while on-treatment or an improvement from baseline. Patients with MMR or better are counted as CCyR if a valid cytogenetic assessment is not available on a specific date. Associated two-sided 95% CI based on the exact method by Clopper–Pearson.

*CCyR* complete cytogenetic response, *CI* confidence interval, *CML* chronic myeloid leukemia, *CP* chronic phase, *MCyR* major cytogenetic response, *Ph* Philadelphia chromosome, *TKI* tyrosine kinase inhibitor.

**Fig. 2:**
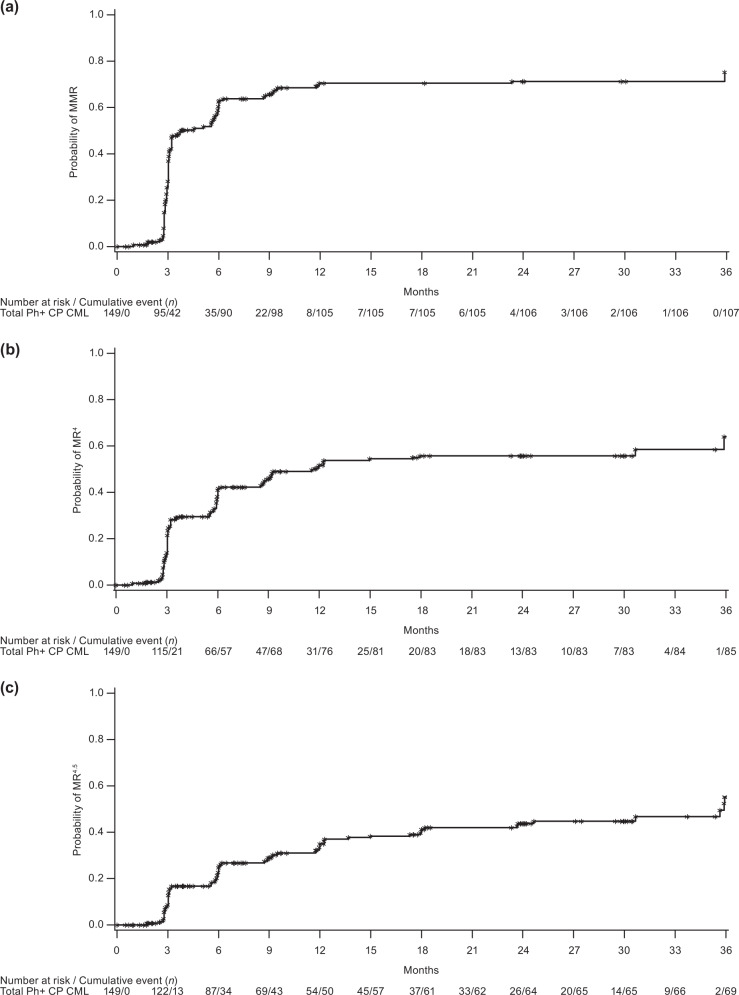
Cumulative Incidence of Molecular Response in Patients with Ph+ CP CML. (**a**) MMR, (**b**) MR^4^, and (**c**) MR^4.5^. *CML* chronic myeloid leukemia, *CP* chronic phase, *MMR* major molecular response, *MR* molecular response, *Ph* Philadelphia chromosome.

The cumulative MMR rate by 1 year was 70.5% in the overall Ph+ CP CML cohort (TKI-resistant: 60.5%; TKI-intolerant: 80.8%); rates according to line of therapy are shown in Table 3. In patients without MMR at baseline, the cumulative MMR rate by 1 year was 58.2% (TKI-resistant: 43.8%; TKI-intolerant: 80.6%). (Table 3). By 1 year, cumulative MR^4^ and MR^4.5^ rates, respectively, were 51.0 and 33.6% (TKI-resistant: 39.5 and 25.0%; TKI-intolerant: 63.0 and 42.5%). In patients without the respective response at baseline, cumulative MR^4^ and MR^4.5^ rates were 42.0 and 26.7% (TKI-resistant: 26.7 and 20.8%; TKI-intolerant: 59.6 and 33.9%). At any time, respective cumulative MR^4^ and MR^4.5^ rates were 57.0% and 46.3% and 49.1% and 40.5% in patients without the respective baseline response. Cumulative MR^4^ and MR^4.5^ rates at any time across therapy lines and in TKI-resistant and TKI-intolerant patients are shown in Table 3. Responding patients typically achieved MMR within 1 year of bosutinib initiation and a deep MR within 2 years of bosutinib initiation, although a small proportion of patients achieved MR at later time points (Fig. 2). Of three patients with Ph−/*BCR-ABL1*+ CML, one each had MMR, *BCR-ABL1* IS ≤ 1%, and no response.

**Table 3:** Cumulative molecular response rates by 1 year, by 2 years, and any time on treatment in patients with Ph + CP CML: total cohort, by line of therapy and by TKI resistance or intolerance (overall and excluding patients with the respective baseline response).

	Total *N* = 156	By line of therapy			By TKI resistance or intolerance	
		Second-line *n* = 46	Third-line *n* = 61	Fourth-line *n* = 49	Resistant *n* = 83	Intolerant *n* = 73
Cumulative molecular response, % (95% CI)						
Evaluable patients, *n*	149	46	55	48	76	73
MMR						
By 1 year	70.5 (62.5–77.7)	80.4 (66.1–90.6)	74.5 (61.0–85.3)	56.3 (41.2–70.5)	60.5 (48.6–71.6)	80.8 (69.9–89.1)
By 2 years	71.1 (63.2–78.3)	82.6 (68.6–92.2)	74.5 (61.0–85.3)	56.3 (41.2–70.5)	61.8 (50.0–72.8)	80.8 (69.9–89.1)
Any time on treatment	71.8 (63.9–78.9)	82.6 (68.6–92.2)	76.4 (63.0–86.8)	56.3 (41.2–70.5)	61.8 (50.0–72.8)	82.2 (71.5–90.2)
MR^4^						
By 1 year	51.0 (42.7–59.3)	58.7 (43.2–73.0)	54.5 (40.6–68.0)	39.6 (25.8–54.7)	39.5 (28.4–51.4)	63.0 (50.9–74.0)
By 2 years	55.7 (47.3–63.8)	67.4 (52.0–80.5)	60.0 (45.9–73.0)	39.6 (25.8–54.7)	46.1 (34.5–57.9)	65.8 (53.7–76.5)
Any time on treatment	57.0 (48.7–65.1)	69.6 (54.2–82.3)	61.8 (47.7–74.6)	39.6 (25.8–54.7)	46.1 (34.5–57.9)	68.5 (56.6–78.9)
MR^4.5^						
By 1 year	33.6 (26.0–41.7)	32.6 (19.5–48.0)	36.4 (23.8–50.4)	31.3 (18.7–46.3)	25.0 (15.8–36.3)	42.5 (31.0–54.6)
By 2 years	43.0 (34.9–51.3)	47.8 (32.9–63.1)	45.5 (32.0–59.4)	35.4 (22.2–50.5)	35.5 (24.9–47.3)	50.7 (38.7–62.6)
Any time on treatment	46.3 (38.1–54.7)	56.5 (41.1–71.1)	47.3 (33.7–61.2)	35.4 (22.2–50.5)	36.8 (26.1–48.7)	56.2 (44.1–67.8)
Cumulative molecular response in patients without the respective baseline response, % (95% CI)						
Evaluable patients, *n*	79	25	28	26	48	31
MMR						
By 1 year	58.2 (46.6–69.2)	72.0 (50.6–87.9)	64.3 (44.1–81.4)	38.5 (20.2–59.4)	43.8 (29.5–58.8)	80.6 (62.5–92.5)
By 2 years	59.5 (47.9–70.4)	76.0 (54.9–90.6)	64.3 (44.1–81.4)	38.5 (20.2–59.4)	45.8 (31.4–60.8)	80.6 (62.5–92.5)
Any time on treatment	59.5 (47.9–70.4)	76.0 (54.9–90.6)	64.3 (44.1–81.4)	38.5 (20.2–59.4)	45.8 (31.4–60.8)	80.6 (62.5–92.5)
Evaluable patients, *n*	112	37	38	37	60	52
MR^4^						
By 1 year	42.0 (32.7–51.7)	51.4 (34.4–68.1)	44.7 (28.6–61.7)	29.7 (15.9–47.0)	26.7 (16.1–39.7)	59.6 (45.1–73.0)
By 2 years	48.2 (38.7–57.9)	62.2 (44.8–77.5)	52.6 (35.8–69.0)	29.7 (15.9–47.0)	35.0 (23.1–48.4)	63.5 (49.0–76.4)
Any time on treatment	49.1 (39.5–58.7)	64.9 (47.5–79.8)	52.6 (35.8–69.0)	29.7 (15.9–47.0)	35.0 (23.1–48.4)	65.4 (50.9–78.0)
Evaluable patients, *n*	131	42	46	43	72	59
MR^4.5^						
By 1 year	26.7 (19.4–35.2)	26.2 (13.9–42.0)	28.3 (16.0–43.5)	25.6 (13.5–41.2)	20.8 (12.2–32.0)	33.9 (22.1–47.4)
By 2 years	37.4 (29.1–46.3)	42.9 (27.7–59.0)	39.1 (25.1–54.6)	30.2 (17.2–46.1)	31.9 (21.4–44.0)	44.1 (31.2–57.6)
Any time on treatment	40.5 (32.0–49.4)	52.4 (36.4–68.0)	39.1 (25.1–54.6)	30.2 (17.2–46.1)	33.3 (22.7–45.4)	49.2 (35.9–62.5)

Evaluable molecular population. To be considered a responder, the patient must have maintenance of baseline response while on-treatment or an improvement from baseline. MMR: *BCR-ABL1* IS ≤ 0.1%; MR^4^: *BCR-ABL1* IS ≤ 0.01%; MR^4.5^: *BCR-ABL1* IS ≤ 0.0032%. Associated two-sided 95% CI based on the exact method by Clopper–Pearson.

*CI* confidence interval, *CML* chronic myeloid leukemia, *CP* chronic phase, *IS* international scale, *MMR* major molecular response, *MR* molecular response, *Ph* Philadelphia chromosome, *TKI* tyrosine kinase inhibitor.

Eleven patients with Ph+ CP CML had mutations at baseline; of these, two achieved MR^5^, one achieved MR^4.5^, one achieved MMR, and four achieved CHR as best response. Molecular responses were observed in patients with F359I, Y253F, A365V, and E255V mutations (Supplementary Table S1). Of 20 patients with Ph+ CP CML evaluated for new *BCR-ABL1* point mutations, one patient in the third-line cohort with a baseline Y253F mutation had a newly detectable T315I mutation.

By the cutoff date, no patient with Ph+ CP CML had progressed to AP/BP on treatment. After a median follow-up of 30.4 months (range 0.7–44.6), 1- and 2-year Kaplan–Meier OS rates, respectively, were 98.0% and 96.0%, for patients with Ph+ CP CML (second-line: 100 and 97.7%; third-line: 96.7 and 95.0%; fourth-line: 97.9 and 95.4%; Supplementary Fig. S2A). The respective rates were 97.5 and 94.9% in TKI-resistant and 98.6 and 97.2% in TKI-intolerant patients (Supplementary Fig. S2B). After a median follow-up of 20.6 months (range 1.6–32.3) and 26.5 months (3.5–41.4) for patients with Ph+ AP CML and Ph− CML, respectively, OS rates (95% CI) at both 1 year and 2 years were 100% (100–100%) and 66.7% (5.4–94.5%).

### Safety

In the overall patient population (*N* = 163), 99.4% of patients had ≥1 any grade TEAE and 73.6% of patients had ≥1 grade 3/4 TEAE. Treatment-emergent serious AEs were reported in 35.6% of patients. TEAEs led to dose reduction and temporary discontinuation in 77.3% and 75.5% of patients, respectively, and 42 (25.8%) discontinued treatment due to AEs. The most common AEs leading to discontinuation (≥2% of patients) were increased alanine aminotransferase (4.9%) and increased aspartate aminotransferase (2.5%). In TKI-resistant and TKI-intolerant patients, respectively, rates of any grade TEAEs were 100% and 98.6%, and rates of grade 3/4 TEAEs were 69.9% and 79.5%. The rate of dose reductions due to TEAEs was 73.5% in TKI-resistant patients and 84.9% in TKI-intolerant patients; respective rate of temporary discontinuations due to TEAEs was 68.7% and 84.9%. Overall, 21.7% and 28.8% of TKI-resistant and TKI-intolerant patients, respectively, discontinued treatment due to AEs. There were no relevant differences in the overall frequency of TEAEs, grade 3/4 TEAEs, or dose reductions/temporary discontinuations due to TEAEs across lines of treatment.

The most common TEAEs (>30%) in the overall patient population were diarrhea (87.7%), nausea (39.9%), and vomiting (32.5%) (Table 4). However, only two (1.2%), three (1.8%), and two (1.2%) patients discontinued due to diarrhea, nausea, and vomiting, respectively. Median (range) time to first TEAE of diarrhea was 2.0 (1–304) days, and the median (range) duration of diarrhea event (any grade) was 8.0 (1–715) days. Grade 3/4 TEAEs occurring in >5% of patients were diarrhea (16.0%), increased alanine aminotransferase (14.1%), thrombocytopenia (8.0%), increased lipase (6.7%), and pleural effusion (6.1%). TEAEs of special interest included cardiac (14.7%), vascular (11.7%), effusion (18.4%), metabolic (8.0%), and gastrointestinal (91.4%; Table 5).

**Table 4:** Summary of TEAEs (all grade TEAEs reported in ≥10% of patients).

*n* (%)	Total (*N* = 163)	
	All grades	Grades 3/4
**Any TEAE**	**162** (**99.4)**	**120 (73.6)**
Diarrhea	143 (87.7)	26 (16.0)
Nausea	65 (39.9)	4 (2.5)
Vomiting	53 (32.5)	6 (3.7)
Abdominal pain	46 (28.2)	7 (4.3)
Headache	45 (27.6)	1 (0.6)
ALT increased	42 (25.8)	23 (14.1)
Fatigue	39 (23.9)	2 (1.2)
Abdominal pain upper	36 (22.1)	2 (1.2)
Dyspnea	35 (21.5)	5 (3.1)
Asthenia	33 (20.2)	4 (2.5)
AST increased	32 (19.6)	7 (4.3)
Cough	30 (18.4)	1 (0.6)
Pyrexia	29 (17.8)	5 (3.1)
Constipation	28 (17.2)	2 (1.2)
Arthralgia	28 (17.2)	1 (0.6)
Pleural effusion	27 (16.6)	10 (6.1)
Back pain	27 (16.6)	4 (2.5)
Anemia	25 (15.3)	7 (4.3)
Rash	25 (15.3)	7 (4.3)
Dizziness	25 (15.3)	0
Blood creatinine increased	24 (14.7)	0
Nasopharyngitis	24 (14.7)	0
Lipase increased	23 (14.1)	11 (6.7)
Myalgia	22 (13.5)	2 (1.2)
Decreased appetite	22 (13.5)	1 (0.6)
Edema peripheral	22 (13.5)	1 (0.6)
Thrombocytopenia	18 (11.0)	13 (8.0)
Pain in extremity	17 (10.4)	2 (1.2)
Pruritus	17 (10.4)	2 (1.2)

Full analysis set. Classification of adverse events is based on the Medical Dictionary for Regulatory Activities (v21.1).

*ALT* alanine aminotransferase, *AST* aspartate aminotransferase, *CML* chronic myeloid leukemia, *TEAE* treatment-emergent adverse event.

**Table 5:** TEAEs of special interest.

*n* (%)	Total (*N* = 163)
**Cardiac TEAEs**	
Any TEAE	24 (14.7)
Cardiac disorders	23 (14.1)
Cardiac failure	6 (3.7)
Atrial fibrillation	5 (3.1)
Tachycardia	3 (1.8)
Arrhythmia	2 (1.2)
Bradycardia	2 (1.2)
Cardiac failure congestive	2 (1.2)
Atrial flutter	1 (0.6)
Atrioventricular block complete	1 (0.6)
Bundle branch block right	1 (0.6)
Cardiac failure acute	1 (0.6)
Cardiac flutter	1 (0.6)
Cardiogenic shock	1 (0.6)
Extrasystoles	1 (0.6)
Sinus bradycardia	1 (0.6)
Investigations	1 (0.6)
Electrocardiogram QT interval prolonged	1 (0.6)
**Vascular TEAEs**	
Any TEAE	19 (11.7)
Cardiovascular	5 (3.1)
Angina pectoris	2 (1.2)
Angina unstable	1 (0.6)
Coronary artery occlusion	1 (0.6)
Myocardial ischemia	1 (0.6)
Cerebrovascular	5 (3.1)
Cerebrovascular accident	2 (1.2)
Transient ischemic attack	2 (1.2)
Carotid artery stenosis	1 (0.6)
Peripheral vascular	10 (6.1)
Peripheral arterial occlusive disease	3 (1.8)
Peripheral ischemia	2 (1.2)
Aortic stenosis	1 (0.6)
Arterial rupture	1 (0.6)
Intermittent claudication	1 (0.6)
Peripheral coldness	1 (0.6)
Vascular pain	1 (0.6)
**Effusion TEAEs**	
Any TEAE	30 (18.4)
Pleural effusion	27 (16.6)
Pericardial effusion	8 (4.9)
**Metabolic TEAEs**	
Any TEAE	13 (8.0)
Hyperglycemia	5 (3.1)
Diabetes mellitus	4 (2.5)
Hypercholesterolemia	3 (1.8)
Hypertriglyceridemia	3 (1.8)
Hyperlipidemia	1 (0.6)
**Gastrointestinal TEAEs**	
Any TEAE	149 (91.4)
Diarrhea	143 (87.7)
Abdominal pain	67 (41.1)
Nausea	65 (39.9)
Vomiting	53 (32.5)
Constipation	28 (17.2)

Full analysis set. Classification of adverse events is based on the Medical Dictionary for Regulatory Activities (v21.1). See Supp. Methods for adverse events of special interest cluster definitions.

Totals for the number of patients at a higher level are not necessarily the sum of those at the lower levels since a patient may report two or more different TEAEs within the higher level category.

*TEAE* treatment-emergent adverse events.

There were 12 deaths, seven of which occurred within 28 days of last dose (six due to AEs not related to bosutinib and one due to CML, as determined by the investigator) and five deaths occurred beyond 28 days of last dose (four due to AEs not related to bosutinib and one due to an unknown cause, as determined by the investigator). AEs resulting in death were: acute kidney injury, respiratory insufficiency due to aspiration, cerebral tumor, chronic briden-ileus, hemorrhagic shock, lymphoma, metastatic lung cancer, multiorgan failure, prostate adenocarcinoma, and sepsis.

### PROs

**Fig. 3:**
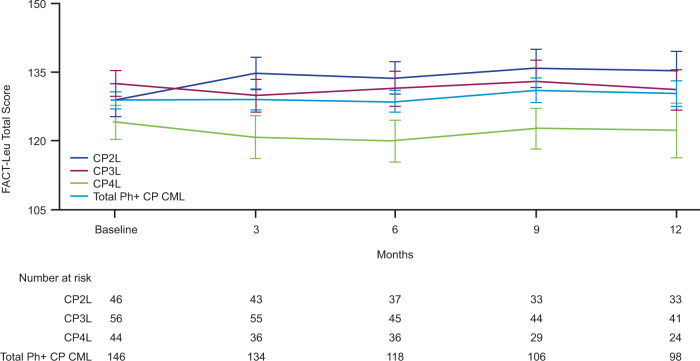
Observed mean (SE) FACT-Leu values over 12 months of bosutinib treatment. *CML* chronic myeloid leukemia, *CP* chronic phase, *CP2L* second-line, *CP3L* third-line, *CP4L* fourth-line, *FACT-Leu* functional assessment of cancer therapy–leukemia, *Ph* Philadelphia chromosome, *SE* Standard error.

At baseline, total FACT-Leu scores were similar (<5% difference) between the second- and third-line cohorts; slightly lower scores were reported in the fourth-line cohort at baseline (Fig. 3 and Supplementary Table S2). Total FACT-Leu scores were maintained from baseline in all cohorts following 12 months of bosutinib treatment (Fig. 3); additionally, at month 12, no mean change in any individual FACT-Leu domain score from baseline met the MID, indicating preservation of health-related QoL (HRQoL) (Supplementary Fig. S3; Fig. 3). Within these MIDs, FACT-Leu scores increased slightly from baseline to month 12 in the second-line cohort and (except for emotional well-being) decreased slightly in the third-line cohort; positive versus negative changes were less consistent in the fourth-line cohort. The effect of MR on HRQoL was variable. For patients who achieved MR^5^, the leukemia-specific domain showed the greatest improvement, with a large effect size, followed by the emotional well-being domain and TOI FACT-Leu, with medium effect sizes; the social well-being domain was the only domain to demonstrate a beyond-trivial reduction in HRQoL, with a medium effect size (Supplementary Fig. S4).

## Discussion

Overall, data from the phase 4 BYOND study confirm the efficacy findings from the phase 1/2 study of bosutinib in the second-, third- and fourth-line settings [13, 16–20]. High rates of cytogenetic and molecular responses were observed across patients with Ph+ CP CML treated with bosutinib, including a large proportion of patients who achieved deep MR. The majority of patients achieved deeper responses relative to baseline with bosutinib, even though most patients entered the study with at least MCyR from prior treatment. These high response rates were seen in TKI-resistant and TKI-intolerant patients and across treatment lines. Although patients with a more resistant phenotype showed lower response rates compared with TKI-intolerant patients, responses were also seen in patients with resistance to imatinib or the 2nd-generation TKIs dasatinib and nilotinib.

Data in patients with CML resistant/intolerant to prior TKIs were also reported in studies of nilotinib, dasatinib, and ponatinib [11, 14, 21]. In a phase 2 study of second-line nilotinib 400 mg twice daily in imatinib-resistant/intolerant patients (*N* = 321), MCyR, CCyR, and MMR rates at any time on treatment were 59%, 44%, and 28%, respectively, after a minimum follow-up of 24 months [21]. In a phase 3 study of second-line dasatinib (multiple doses) in imatinib-resistant/intolerant patients (*N* = 670), MCyR, CCyR and MMR rates by 24 months were 61–63%, 50–54%, and 37–38%, respectively [11]. In the present study, second-line bosutinib yielded MCyR, CCyR, and MMR rates (both by 24 months and at any time) of 80.0%, 81.3%, and 76.0%, respectively, in patients without the respective baseline response. For bosutinib, achieved rates are used for the purpose of comparisons with the nilotinib and dasatinib studies due to the higher proportion of patients with baseline MCyR enrolled in BYOND: 77.8% versus 11% and 14–20% for nilotinib and dasatinib, respectively [11, 21, 22]. In the present study, response rates were higher in patients treated with fewer previous TKIs; however, response was also seen in heavily pretreated patients: 47.1% and 38.5% of patients (without the respective baseline response) treated with bosutinib fourth-line therapy achieved CCyR and MMR, respectively, by 1 year. These data are comparable to those reported in a phase 2 study of ponatinib 45 mg QD in heavily pretreated patients with CP CML without the T315I mutation (*N* = 203); after a median follow-up of 15 months, MCyR, CCyR, and MMR rates by 12 months were 51%, 40%, and 27%, respectively [14]. In summary, the current data for bosutinib from BYOND, with a median follow-up of 30.4 months, showed comparable cytogenetic and molecular response rates to those reported with nilotinib, dasatinib, or ponatinib treatment.

Overall, AEs with bosutinib were manageable. The reported AEs were consistent with the known safety profile of bosutinib and no new safety issues were identified [6, 17–20, 23–27]. Patients intolerant to previous therapies had a slightly higher incidence of grade 3/4 TEAEs and required more frequent dose adjustments to manage AEs than TKI-resistant patients. Nevertheless, most patients intolerant to previous TKIs, including patients intolerant to all prior TKIs, were able to remain on treatment with bosutinib (median treatment duration, 25.3 months). The overall discontinuation rate due to AEs was consistent with the previous phase 1/2 study, despite approximately half of patients being intolerant to all prior TKI therapy, which indicates that, in general, AEs were manageable with dose reductions and temporary discontinuations. Despite a high incidence of diarrhea, which is usually transient and often improves with dietary changes and the administration of supportive care, bosutinib discontinuation due to this AE was low [28]. In patients who have an increase in transaminases, it is advisable to avoid other hepatotoxic drugs and excess alcohol consumption, and monitor liver enzymes more frequently; dose modifications and/or discontinuation may be required in more severe cases [29].

As with imatinib, patients treated with dasatinib or nilotinib may eventually develop resistance to treatment. In addition, some patients may be unable to continue treatment with dasatinib, nilotinib, or ponatinib due to intolerance, or the safety profiles of these agents may preclude their use in patients with certain comorbidities. Safety comparisons across TKI studies are limited; however, the varying “off target” effects of bosutinib, nilotinib, dasatinib, and ponatinib are reflected in the safety of these agents, with each BCR-ABL1 TKI showing a distinct toxicity profile.

Vascular AEs have been described mainly with nilotinib and ponatinib. Metabolic AEs, potentially contributing to vascular toxicity, are also frequently reported with nilotinib [14, 21, 30]. The European LeukemiaNet (ELN) recommendations, therefore, state that nilotinib is contraindicated in patients with a history of coronary heart disease, cerebrovascular accidents, or peripheral arterio-occlusive disease and that previous or concomitant arteriovascular disease is a contraindication to ponatinib in second- or third-line treatment. Pulmonary toxicities, such as pleural effusion and more rarely pulmonary arterial hypertension, have been primarily associated with dasatinib treatment [11, 31–33], and the ELN panel recommended to avoid the use of dasatinib in patients with respiratory failure and previous or concomitant pleuro-pulmonary or pericardial disease. While the incidence of these specific AEs with bosutinib in BYOND was higher than previously reported [13, 16, 34, 35], the heavily pretreated nature of the patients might have contributed to this. ELN recommendations state that no relevant comorbidities or contraindications have been identified for bosutinib [15], and bosutinib is a treatment option for patients with pulmonary or cardiovascular comorbidities, diabetes mellitus, or hyperglycemia due to the lower risk of these types of events [36]. Therefore, bosutinib is an appropriate treatment option for patients resistant or intolerant to prior TKIs, including in patients who have previously received treatment with a 2nd-generation TKI and in those who present with multiple comorbidities.

HRQoL was maintained from baseline in patients with CP CML following 12 months of bosutinib treatment. Changes from baseline in patient-reported outcomes measures at month 12 were comparable to those observed in previously treated patients in the initial phase 1/2 study of bosutinib, wherein long-term efficacy and HRQoL stability were reported [19, 37, 38]. In addition, FACT-G scores in the current study were consistent with those previously reported for bosutinib in newly diagnosed patients with CML, in the general population, as well as in patients with various cancers [39–43]. Maintenance of HRQoL is important for patients with CP CML who potentially will receive lifelong TKI treatment, and PRO results from this phase 4 study suggest bosutinib is a treatment option with manageable AEs, providing further support for its use in patients with CP CML resistant/intolerant to prior TKIs. The impact of clinical improvement on different dimensions of HRQoL was variable; for the majority of domains, a deeper MR was associated with better HRQoL.

In summary, high rates of cytogenetic and molecular responses, including a large proportion of patients who achieved MR^4^ and MR^4.5^, were observed with bosutinib treatment. AEs that occurred with bosutinib were manageable [28, 29], further evidenced by maintenance of HRQoL, and the reported AEs were consistent with the known safety profile of bosutinib. The results from this phase 4 study further confirm the use of bosutinib for patients with CML resistant/intolerant to prior TKIs across all treatment lines.

### Supplementary information

suppl. material
