# Supplementary material for: Bosutinib for pretreated patients with chronic phase chronic myeloid leukemia: primary results of the phase 4 BYOND study
Source: Leukemia. 2020 Jun 22;34(8):2125–37. doi: 10.1038/s41375-020-0915-9 (PMC7387243; doi:10.1038/s41375-020-0915-9)
Supplement: Supplementary file 1 — suppl. material [file 41375_2020_915_MOESM1_ESM.pdf]

Supplementary Methods

Study design and patients

Patients had to have adequate bone marrow function, defined as absolute neutrophil count (ANC) >1 x 10^9/L and platelets ≥75 x 10^9/L for patients with chronic phase chronic myeloid leukemia (CP CML) after one or two prior tyrosine kinase inhibitors (TKIs), and ANC >0.5 x 10^9/L and platelets ≥50 x 10^9/L for patients with accelerated/blast phase (AP/BP) CML or CP CML after three prior TKIs. Patients also had to have adequate hepatic and renal function, defined as: aspartate (AST) and alanine (ALT) aminotransferase ≤2.5 x the upper limit of normal (ULN) or ALT/AST ≤5 x ULN if attributable to liver involvement of leukemia; total bilirubin ≤1.5 x ULN (unless the bilirubin was principally unconjugated and there was a strong suspicion of subclinical hemolysis, or the patient had documented Gilbert's Disease); alkaline phosphatase ≤2.5 x ULN; and creatinine ≤1.5 x ULN or estimated creatinine clearance ≥60 mL/min.

For non-hematologic adverse events (AEs), the following dose adjustments were permitted. Patients who experienced a grade 1 AE were to remain on the current dose level. For grade 2 AEs, bosutinib treatment was to be interrupted, and reintroduced at the same dose or the dose reduced by one level upon recovery to grade ≤1 within 4 weeks of stopping treatment. For grade 3 AEs, bosutinib treatment was to be interrupted, the dose reduced by one level upon recovery to grade ≤1 within 4 weeks of stopping treatment. If recovery was >4 weeks, the patient was to be evaluated to determine if bosutinib treatment should continue. Bosutinib treatment was to be discontinued in the event of a grade 4 AE, and the patient was to be evaluated to determine if bosutinib treatment should continue with an appropriate dose reduction. For grade 3/4 diarrhea, bosutinib treatment was to be interrupted and then resumed at 400 mg once daily upon recovery to grade ≤1.

For hematologic AEs, the following dose adjustments were permitted. Patients who experienced a grade 1 or grade 2 AE were to remain on the current dose level. For grade 3 AEs, bosutinib was to be interrupted. If recovered to grade ≤2 within 2 weeks, bosutinib was to be re-introduced at the same dose. If recovered within 4 weeks, bosutinib was to be reduced by one dose level. In the case of recurrent grade 3 toxicity, the dose was to be reduced upon recovery to grade ≤2. If recovery was >4 weeks, the patient was to be evaluated to determine if bosutinib treatment should continue. Bosutinib treatment was to be discontinued in the event of a grade 4 AE, and the patient was to be evaluated to determine if bosutinib treatment should continue with an appropriate dose reduction.

Endpoints and analyses

Molecular, cytogenetic, and hematologic response

Analyses of hematologic and cytogenetic response were based on data from local laboratory assessments. Real-time quantitative polymerase chain reaction for molecular response assessment (*BCR-ABL1* transcript levels international scale [IS]) and mutational analysis of the *BCR-ABL1* kinase domain were performed by a central laboratory. Hematologic assessments were performed at baseline and every week until week 4, at week 8, every 3 months until week 52, then at 6-month intervals during years 2, 3 and 4, and at end of treatment. Cytogenetic and molecular response assessments were performed at baseline and every 3 months until week 52, then at 6-month intervals

during years 2, 3 and 4, and at end of treatment. All assessments were performed in the event of treatment failure and/or disease progression. Mutational analyses were performed from the peripheral blood or bone marrow samples that were used for molecular response assessment.

Resistance and intolerance

Patients were categorized as resistant or intolerant to prior TKIs by the investigator. Resistance was defined in accordance with European LeukemiaNet (ELN) 2013 recommendations [1] or National Comprehensive Cancer Network (NCCN) guidelines [2]. Intolerance to prior TKIs was defined as ≥1 of the following criteria: any life-threatening grade 4 non-hematologic toxicity; any grade 3/4 non-hematologic toxicity that persisted despite dose reduction and optimal symptomatic measures; grade 3/4 hematologic toxicity that was unresponsive to supportive measures and required dose reduction below the accepted minimal effective dose; or any combination of non-hematologic toxicities of any grade that persisted despite supportive measures and necessitated a change of therapy.

Treatment-emergent adverse events

Medical Dictionary for Regulatory Activities (MedDRA) preferred terms included in treatment-emergent AE (TEAE) clusters of special interest:

- Cardiac: high level group term (HLGT) in cardiac arrhythmias, heart failure; preferred term (PT) in cardiac death, sudden cardiac death, sudden death, ejection fraction decreased; standardized MedDRA query (SMQ) Torsade de pointes/QT prolongation (narrow).
- Effusions: PT in pleural effusion or pericardial effusion.
- Gastrointestinal:
    - Abdominal pain: PT in abdominal pain, abdominal pain upper, abdominal pain lower, gastrointestinal pain
    - Diarrhea: PT in diarrhea, defecation urgency, frequent bowel movements
    - Vomiting: PT in vomiting, vomiting projectile, regurgitation, retching.
- Metabolic:
    - Diabetes mellitus: HLT diabetes mellitus (including subtypes)
    - Hypercholesterolemia: PT in hypercholesterolemia, blood cholesterol increased
    - Hyperglycemia: PT in hyperglycemia, blood glucose increased
    - Hyperlipidemia: PT in hyperlipidemia, lipids increased
    - Hypertriglyceridemia: PT in hypertriglyceridemia, blood triglycerides increased.
- Vascular:
    - Cardiovascular: HLGT in coronary artery disorders; high level term (HLT) in arterial therapeutic procedures (excluding aortic), vascular imaging procedures not elsewhere classified (NEC), vascular therapeutic procedures NEC; PT in transcatheter arterial chemoembolization
    - Cerebrovascular: HLT in central nervous system hemorrhages and cerebrovascular accidents, central nervous system vascular disorders NEC, transient cerebrovascular events; PT in subarachnoid hemorrhage
    - Peripheral vascular: HLGT in arteriosclerosis, stenosis, vascular insufficiency and necrosis, embolism and thrombosis; HLT in non-site-specific vascular disorders NEC, peripheral vascular disorders NEC (excluding the PTs flushing and hot flush), PT in intestinal ischemia.

The following MedDRA preferred terms were clustered for cytopenias:

- Anemia: PT in anemia, hemoglobin decreased
- Neutropenia: PT in neutropenia, neutrophil count decreased
- Leukopenia: PT in leukopenia, white blood cell count decreased
- Thrombocytopenia: PT in thrombocytopenia, platelet count decreased.

Patient-reported outcomes

Patient-reported outcomes were assessed using the Functional Assessment of Cancer Therapy-Leukemia (FACT-Leu) quality-of-life (QoL) questionnaire at baseline, every 3 months for the first year, and every 6 months during years 2, 3 and 4 of treatment [3, 4]. Higher scores reflected better QoL. Minimal important differences (MID) were defined as the smallest change in a PRO measure that was perceived by patients as beneficial or would result in a clinician considering change in treatment. MID domain scores included: 2–3 in physical well-being), 2 for emotional well-being, 2–3 in functional well-being, 3–7 for FACT-General (FACT-G), 4–7 in leukemia-specific subscale, 6–12 for FACT-Leu total, and 5–6 for trial outcome index (TOI) FACT-Leu [5]. The MID has not been defined for social well-being.

References

1. Baccarani M, Deininger MW, Rosti G, Hochhaus A, Soverini S, Apperley JF *et al.* European LeukemiaNet recommendations for the management of chronic myeloid leukemia: 2013. Blood 2013; **122:** 872-884.
2. O'Brien S, Berman E, Moore JO, Pinilla-Ibarz J, Radich JP, Shami PJ *et al.* NCCN Task Force report: tyrosine kinase inhibitor therapy selection in the management of patients with chronic myelogenous leukemia. J Natl Compr Canc Netw 2011; **9 Suppl 2:** S1-25.
3. Cella D, Jensen SE, Webster K, Hongyan D, Lai JS, Rosen S, *et al*. Measuring health-related quality of life in leukemia: the Functional Assessment of Cancer Therapy--Leukemia (FACT-Leu) questionnaire. *Value in health: the journal of the International Society for Pharmacoeconomics and Outcomes Research*. 2012; **15**: 1051-1058.
4. FACIT.org. FACIT Questionnaires. 2019 [cited May 4 2020]; Available from: https://www.facit.org/FACITOrg/Questionnaires.
5. Trask PC, Cella D, Besson N, Kelly V, Masszi T, Kim DW. Health-related quality of life of bosutinib (SKI-606) in imatinib-resistant or imatinib-intolerant chronic phase chronic myeloid leukemia. *Leuk Res*. 2012; **36**: 438-442.

Supplementary Fig. S1. Bosutinib Dose Over Time in Patients With Ph+ CP CML

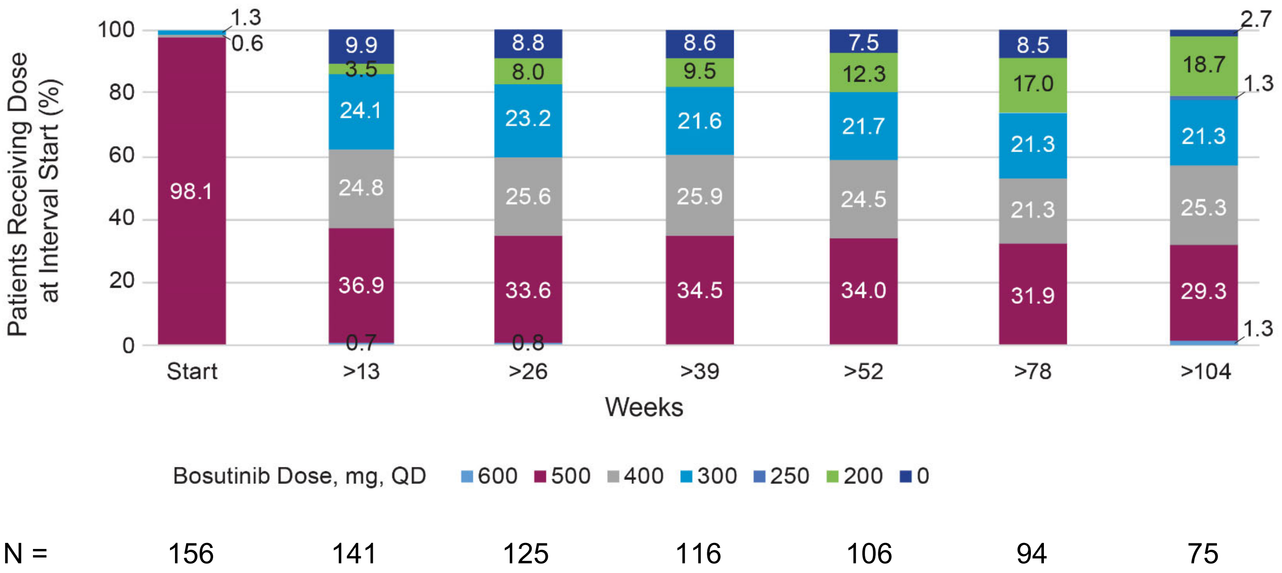

Full analysis set for Ph+ CP CML.
CP CML chronic phase chronic myeloid leukemia, *Ph* Philadelphia chromosome.

Supplementary Fig. S2. Overall Survival in Patients With Ph+ CP CML (A) by Line of Therapy and (B) by TKI Resistance or Intolerance

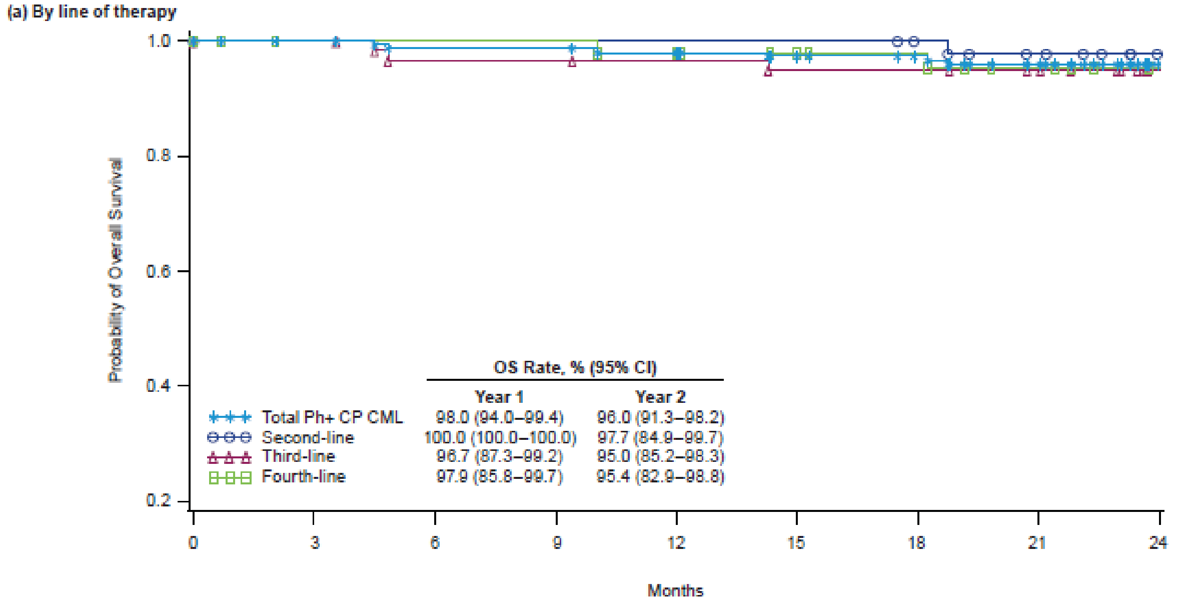

Number at Risk / Cumulative Events (*n*)

	0	3	6	9	12	15	18	21	24
Total Ph+ CP CML	156/0	154/0	151/2	151/2	149/3	143/4	140/4	132/6	115/6
Second-line	46/0	46/0	46/0	46/0	46/0	46/0	44/0	41/1	35/1
Third-line	61/0	61/0	58/2	58/2	57/2	56/3	56/3	54/3	47/3
Fourth-line	49/0	47/0	47/0	47/0	46/1	41/1	40/1	37/2	33/2

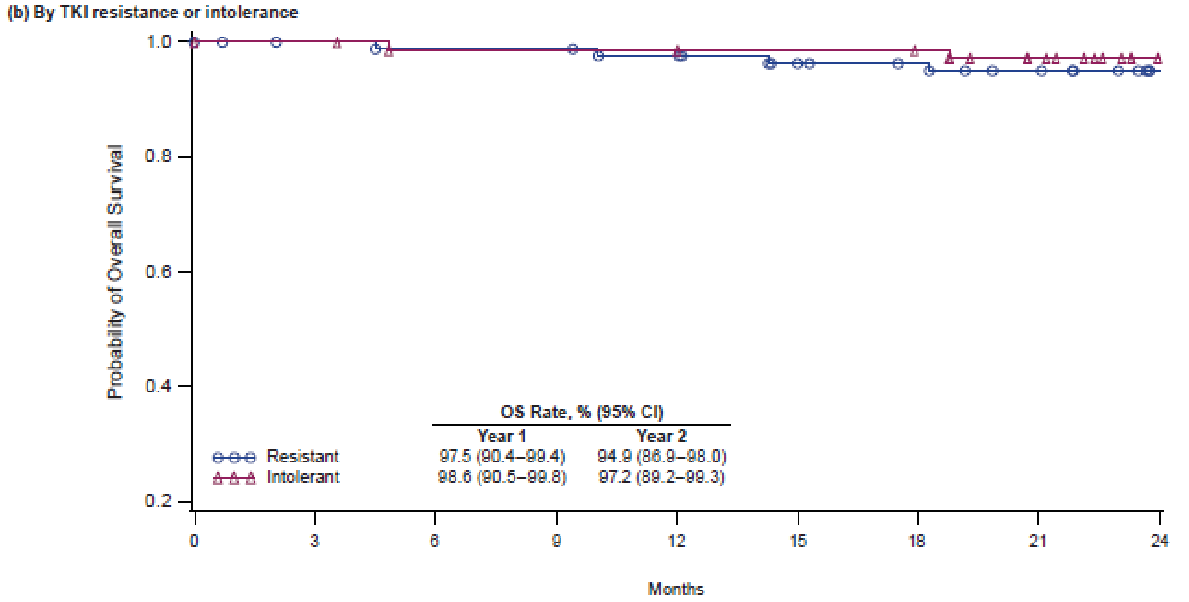

Number at Risk / Cumulative Event (*n*)

	0	3	6	9	12	15	18	21	24
Resistant	83/0	81/0	80/1	80/1	78/2	73/3	71/3	68/4	60/4
Intolerant	73/0	73/0	71/1	71/1	71/1	70/1	69/1	64/2	55/2

Full analysis set for Ph+ CP CML. Open symbols indicate censored observations.
Four deaths occurred after 24 months.
CI confidence interval, *CP CML* chronic phase chronic myeloid leukemia, *Ph* Philadelphia chromosome, *TKI* tyrosine kinase inhibitor.

Supplementary Fig. S3. Mean (95% CI) Changes in FACT-Leu From Baseline Values at Month 12

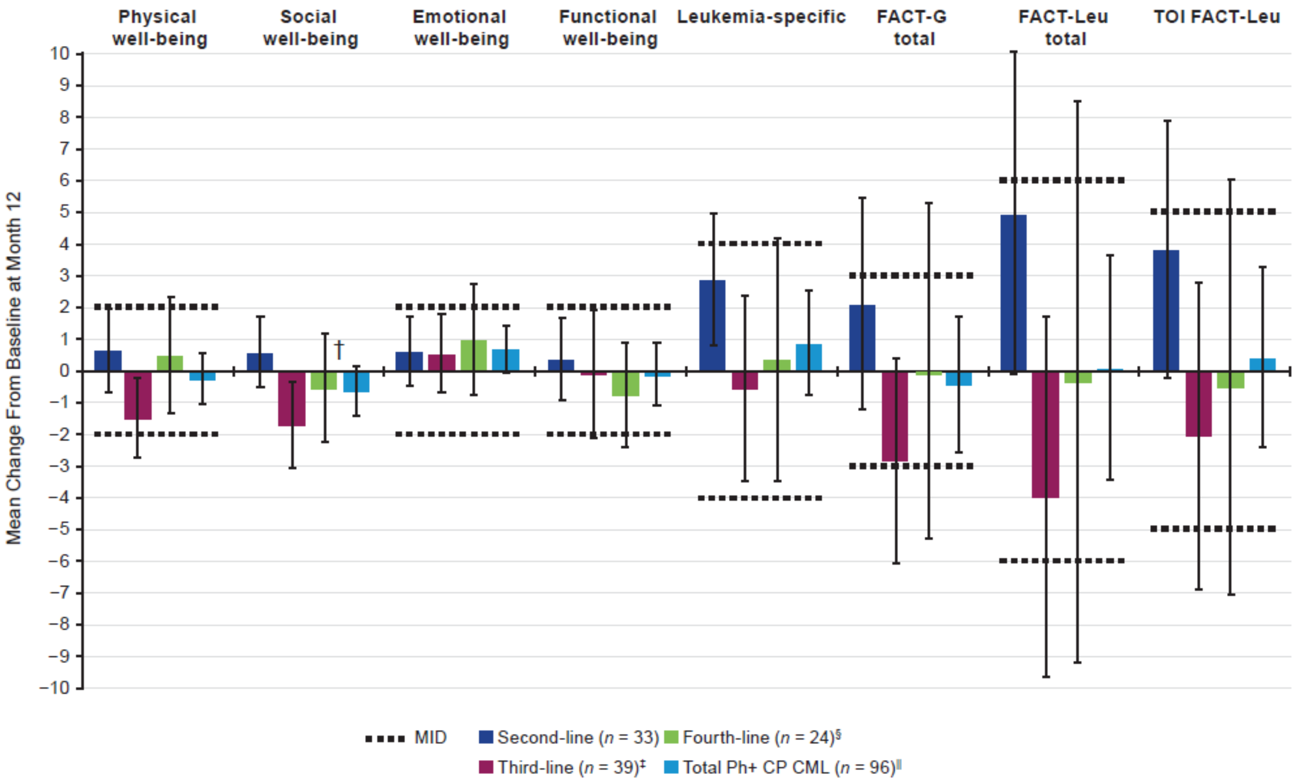

Full analysis set for Ph+ CP CML.
† MID, i.e., the change identified as being clinically meaningful to a patient, has not been defined for social well-being.
‡ n=38 for leukemia-specific and n=37 for FACT-Leu total and TOI FACT-Leu scores; § n=23 for social well-being, FACT-G total, and TOI FACT-Leu and n=22 for FACT-Leu total scores; || n=95 for social well-being, leukemia-specific, and FACT-G total, n=93 for TOI FACT-Leu, and n=92 for FACT-Leu total scores.
CI confidence interval, *CP CML* chronic phase chronic myeloid leukemia, *FACT-G* Functional Assessment of Cancer Therapy–General, *FACT-Leu* Functional Assessment of Cancer Therapy-Leukemia, *MID* minimum important difference, *Ph* Philadelphia chromosome, *TOI* trial outcome index.

Supplementary Fig. S4. Comparison of the Relationships Between Molecular Response and Health-Related Quality of Life (Effect Size†)

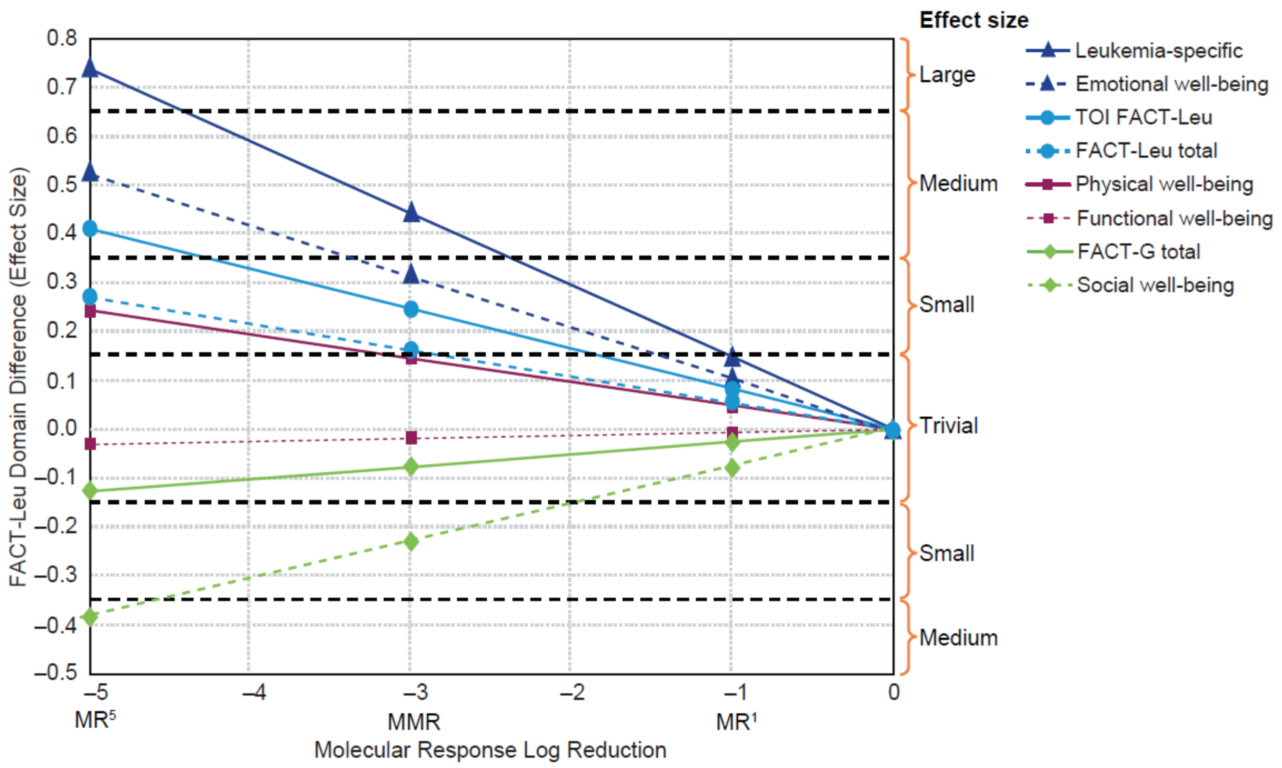

† A (standardized) effect size of 0.2 is considered small (i.e., the difference in means being 0.2 SD unit), 0.5 medium, and 0.8 large; a value of ~0.1 is trivial; midpoints between values of 0.1, 0.2, 0.5, and 0.8 were used to create categorization intervals for effect size.

FACT-G Functional Assessment of Cancer Therapy–General, *FACT-Leu* Functional Assessment of Cancer Therapy–Leukemia, *MMR* major molecular response, *MR* molecular response, *TOI* trial outcome index.

Supplementary Table S1. Response by Baseline Mutational Status in Patients with Ph+ CP CML

Line of therapy	*BCR-ABL1* Mutation	Best Response
Second-line	Y253F	MR^{5}
Second-line	A365V	MR^{5}
Third-line	Y253F[a]	CHR
Third-line	E453K	No response
Third-line	F359I[b]	$MR^{4.5}$
Fourth-line	G250E	CHR
Fourth-line	G250E	No response
Fourth-line	E255K	No response
Fourth-line	E255V	MMR
Fourth-line	Q252H	CHR
Fourth-line	L298V	CHR

Full analysis set for Ph+ CP CML.
[a] One patient with a baseline Y253F mutation had an emergent T315I mutation.
[b] Identified after treatment start on study Day 8.
CHR complete hematologic response, *CP CML* chronic phase chronic myeloid leukemia, *MMR* major molecular response, *MR* molecular response, *Ph* Philadelphia chromosome.

Supplementary Table S2. Baseline FACT-Leu scores

Subscale, mean (SD)	Line of Treatment			Total
	**Second-line (*n* = 46)**	**Third-line (*n* = 58)**	**Fourth-line (*n* = 46)**	**(*N* = 150)**
**Physical well-being**	21.66 (5.51)	22.16 (5.51)	20.28 (6.12)[a]	21.44 (5.72)[b]
**Social well-being**	21.47 (4.74)	22.61 (4.60)	21.43 (4.61)	21.90 (4.65)
**Emotional well-being**	18.50 (3.79)	17.05 (3.99)	16.88 (4.33)	17.44 (4.08)
**Functional well-being**	17.47 (5.84)	18.53 (6.08)	16.81 (6.20)	17.68 (6.05)
**Leukemia-specific**	49.74 (9.04)	51.31 (9.50)[c]	49.27 (9.45)	50.19 (9.32)[b]
**FACT-G total**	79.10 (15.97)	80.35 (13.36)	75.63 (16.29)[a]	78.54 (15.14)[b]
**FACT-Leu total**	128.83 (23.98)	132.42 (20.60)[d]	124.05 (24.02)[e]	128.77 (22.86)[f]
**TOI FACT-Leu**	88.86 (18.27)	92.39 (16.86)[d]	85.74 (19.20)[e]	89.28 (18.12)[f]

Full analysis set for Ph+ CP CML.
CP CML chronic phase chronic myeloid leukemia, *CP2L* second-line, *CP3L* third-line, *CP4L* fourth-line, *FACT-G* Functional Assessment of Cancer Therapy–General, *FACT-Leu* Functional Assessment of Cancer Therapy-Leukemia, *Ph* Philadelphia chromosome, *SD* standard deviation, *TOI* trial outcome index.
[a] *n*=45; [b] *n*=149; [c] *n*=57; [d] *n*=56; [e] *n*=44; [f] *n*=146.
